# Automatic segmentation of coronary angiograms based on fuzzy inferring and probabilistic tracking

**DOI:** 10.1186/1475-925X-9-40

**Published:** 2010-08-20

**Authors:** Zhou Shoujun, Yang Jian, Wang Yongtian, Chen Wufan

**Affiliations:** 1 School of Biomedical Engineering, Southern Medical University, Guangzhou, 510515, China; 2 Department of Optic electronics engineering, Beijing institute of technology, Beijing, 100081, China

## Abstract

**Background:**

Segmentation of the coronary angiogram is important in computer-assisted artery motion analysis or reconstruction of 3D vascular structures from a single-plan or biplane angiographic system. Developing fully automated and accurate vessel segmentation algorithms is highly challenging, especially when extracting vascular structures with large variations in image intensities and noise, as well as with variable cross-sections or vascular lesions.

**Methods:**

This paper presents a novel tracking method for automatic segmentation of the coronary artery tree in X-ray angiographic images, based on probabilistic vessel tracking and fuzzy structure pattern inferring. The method is composed of two main steps: preprocessing and tracking. In preprocessing, multiscale Gabor filtering and Hessian matrix analysis were used to enhance and extract vessel features from the original angiographic image, leading to a vessel feature map as well as a vessel direction map. In tracking, a seed point was first automatically detected by analyzing the vessel feature map. Subsequently, two operators [e.g., a probabilistic tracking operator (PTO) and a vessel structure pattern detector (SPD)] worked together based on the detected seed point to extract vessel segments or branches one at a time. The local structure pattern was inferred by a multi-feature based fuzzy inferring function employed in the SPD. The identified structure pattern, such as crossing or bifurcation, was used to control the tracking process, for example, to keep tracking the current segment or start tracking a new one, depending on the detected pattern.

**Results:**

By appropriate integration of these advanced preprocessing and tracking steps, our tracking algorithm is able to extract both vessel axis lines and edge points, as well as measure the arterial diameters in various complicated cases. For example, it can walk across gaps along the longitudinal vessel direction, manage varying vessel curvatures, and adapt to varying vessel widths in situations with arterial stenoses and aneurysms.

**Conclusions:**

Our algorithm performs well in terms of robustness, automation, adaptability, and applicability. In particular, the successful development of two novel operators, namely, PTO and SPD, ensures the performance of our algorithm in vessel tracking.

## Background

Accurate extraction of the coronary artery tree from coronary angiograms is important for the diagnoses, treatment, and clinical study of various coronary artery diseases. In particular, computer-assisted analysis can improve the performance of quantitative evaluation. It can reduce the inter- and intra-observer variations in determining the severity of coronary artery stenosis [1]. An efficient vessel extraction algorithm also enables the detection of coronary artery motion, as well as the reconstruction of 3D vascular structures from a single-plan or biplane angiographic system [2].

Anatomical structures, such as blood vessels, nerves, and bronchi, present themselves as line-liked structures in 2D images or as tubular structures in 3D images. Over the past 10 years, a variety of approaches have been developed for vessel segmentation from 2D and 3D medical images [3], which typically differ in terms of basic strategies or imaging modalities. Common segmentation approaches employ multiscale filters, morphological segmentations, deformable models, front propagation methods, tracking-based methods, and ridge traversal-based methods. Although many of these approaches are promising for vessel segmentation, developing fully automated, faster, robust, and accurate vessel segmentation algorithms remains highly challenging because of the complexity of vascular structures as well as large variations in image intensities and noise.

Tracking-based methods [4-9] exhibit a natural advantage in extracting arterial axis lines in the angiographic images because of their relative simplicity, as well as their adaptability to variations in vessel diameters. In addition, their ability to capture detailed quantitative descriptions of vessel axes, diameters, and boundaries, leads to high levels of accuracy. Traditional tracking-based methods, however, are guided by simple local features that limit their utility because of difficulties in efficiently integrating advanced features into canonical expressions for guiding the tracking. Therefore, the use of minimum cost functions or multi-scale filters [10-13] to construct the tracking operator poses several advantages in the segmentation of coronary angiograms; this approach, however, may also be problematic when meeting bifurcations or vessel crossings because it can follow only the path with the strongest response.

In this work, we propose a fully automatic tracking-based method that can adapt to varying vessel curvatures and diameters resulting from arterial stenoses or aneurysms. It can also walk across intensity gaps along the longitudinal vessel direction, and manage vessel bifurcations and crossings.

## Methods

**Figure 1:**
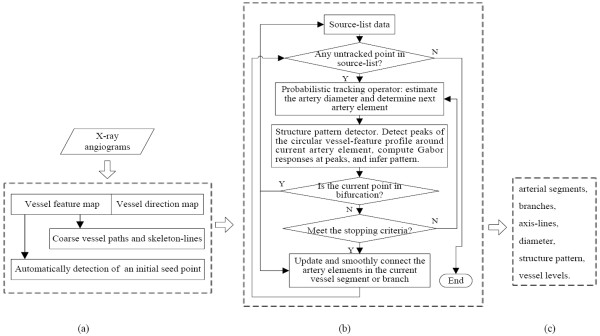
**Overview of the proposed tracking framework for the segmentation of X-ray angiograms with (a) Preprocessing step, (b) Tracking step, and (c) Results**.

An integrated framework was designed to solve the challenging problem of coronary angiogram segmentation. The main components in this framework are summarized in Figure 1. The automatically detected seed point is generally located near the root of the arterial tree or on the main trunk. The probabilistic tracking operator (PTO) was initialized with the initial seed point to search for a candidate artery element with the most similar vessel geometries (including vessel feature, direction, and diameter) in the neighborhood. The vessel structure pattern detector (SPD) was utilized in parallel to classify each detected artery element as bifurcation or crossing. The artery element classified as bifurcation is placed on the list of source data to enable tracking new vessel segments beginning from this artery element in subsequent tracking. The tracking algorithm stops when all points on the list of source data are tracked.

During the process mentioned above, local features and measures, such as the Gabor response, vessel path, skeleton-line, and directional consistency, were also analyzed to integrate the relational vessel attributes for probabilistic vessel tracking and structure pattern identification. The PTO is designed to incorporate local features and measures to determine candidate artery elements along the same vessel segment. These candidates were selected particularly from the overlapping region between the binarized vessel regions and a sampling disk centered at the current artery element under consideration. The size of the sampling disk corresponds to the current vessel diameter, thus rendering the PTO adaptive to varying vessel diameters in case of arterial stenoses or aneurysms. Simultaneously, the SPD works to infer the vessel structure pattern, such as the distal end, segment, bifurcation, and crossing. To complete structure pattern identification, the vessel feature profile scanned from a circle around the current artery element was analyzed and a fuzzy inferring function was applied. In particular, multiple features such as vessel feature, direction continuity, and Gabor responses at the peaks of the vessel feature profile were integrated into the fuzzy inferring function for pattern identification.

### Vessel feature and direction maps

**Figure 2:**
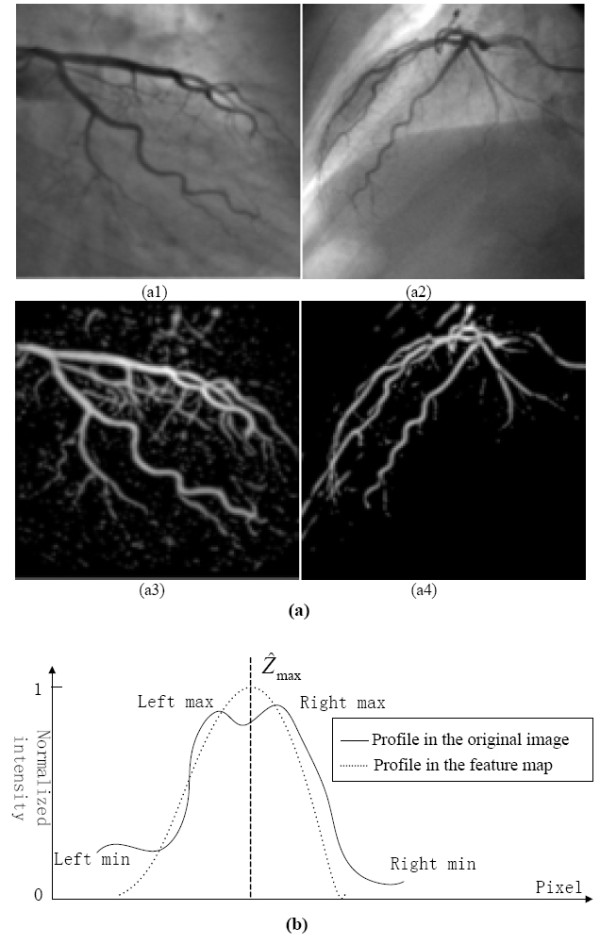
**Show the angiographic images, feature maps and the cross-sectional profiles of coronary artery**. (a1) and (a2) depict two original angiographic images; (a3) and (a4) are their vessel feature maps, respectively. Subfigure (b) depicts the cross-sectional profiles of the vessel in the original image and in the corresponding feature map.

The intensity profile across the longitudinal direction of the vessel may highlight an elevation or even a slight intensity dip at the center, depending on the contrast between vessels and background. On the other hand, the profile along the longitudinal direction of the vessel can be smooth [Figure 2(b)]. Accordingly, the multiscale detection or enhancement of vessel structures can be completed by convolving angiographic image *I*(**x**) [where **x **= (*x*, *y*)] with normalized second-order Gaussian derivatives *G *(**x**; σ*_f_*) at different scales σ*_f _*. The use of a Gaussian filter is important in reducing the influence of noise, properly shaping the line profile and ensuring a large second derivative across the vessel.

In 2D cases, let ∇^2 ^*I *(**x**, σ*_f_*) be a Hessian matrix combined with Gaussian convolution for angiographic image *I*(**x**). It can be written as

(1) $$\nabla^{2}I(\text{x},\sigma_{f})=-\sigma_{f}^{2}\cdot \left\{\left(\begin{array}{cc} \frac{\partial^{2}}{\partial x\partial x} & \frac{\partial^{2}}{\partial x\partial y}\\ \frac{\partial^{2}}{\partial y\partial x} & \frac{\partial^{2}}{\partial y\partial y} \end{array}\right)G(\text{x},\sigma_{f})\right\}*I(\text{x})$$

where *G *(**x**; σ*_f_*) denotes a Gaussian filter with standard deviation σ*_f_*.

The bright line structure on a dark background can be reflected by a Hessian matrix with a large negative eigenvalue λ_1 _and a small eigenvalue λ_2 _of positive or negative sign; that is λ_1 _< 0 and |λ_2_| ≪ |λ_1_|. Various response functions have been proposed in previous studies [11-13]. We provide a similar response function to extract the vessel structures by thresholding the eigenvalue map using a low threshold |*r_λ_*|, expressed in

(2) $$Z(\text{x},\sigma_{f})=\{\begin{array}{ll} \ln(\left|\lambda_{1}(\text{x},\sigma_{f})\right|)+c & \lambda_{1}<r_{\lambda }\\ 0 & \text{else} \end{array}$$

where *c *is a constant. Note that λ_1 _is negative in our case, with $r_{\lambda }=-\sqrt{2\pi \sigma_{f}}$. This response equation provides a vessel discriminant function at scale σ*_f_*. To integrate multiscale responses corresponding to different scales at each location **x**, we select the maximum response across multiple scales, *i.e*.

(3) Z∧(x)=max(Z(x;σf)), σf=σ1,...,σn

where σ_1 _and σ*_n _*are the minimum and maximum scales, determined according to the range of vessel diameters in the angiographic images. In this paper, 14 scales were used. For convenience, the resulting vessel features are normalized to 0[1] in this paper. Vessel feature map $\overset{\wedge }{Z}(\text{x})$ (Figure 2 (a3-a4)) may also be binarized for extracting the vessels, and may further be thinned to obtain the skeletal lines representing the vessels.

The vessel direction map is estimated using the best detected scale. First, the maximum of absolute eigenvalue |λ_1_| is obtained at each location **x **by $\left|\lambda_{opt}(\text{x})|=\max_{\sigma_{f}}|\lambda_{1}(\text{x};\sigma_{f})\right|$. Assuming that the eigenvector corresponds to λ*_opt_*(**x**) is **v***_opt_*(**x**), the direction orthogonal to **v***_opt_*(**x**) is then selected as the vessel direction at **x**. For convenience, we again use **v**_2 _to represent the estimated vessel direction map later in this paper.

Therefore, using this step of multiscale analysis, we obtain three separate maps from the original angiographic images, namely, a vessel feature map, a vessel direction map, and a map of skeleton lines. Next, an initial seed point **x**_0 _is automatically detected from a point within the thinned vessel skeleton-lines, as well as with maximum vessel feature intensity $\overset{\wedge }{Z}({\text{x}}_{0})$. The determined initial seed point is generally located near the root of the arterial tree or the main trunk of the arterial tree. The data above are used for the identification of consequential vessel pattern.

### Probabilistic tracking of coronary artery

A dynamic mechanism was designed for our tracking framework. *First*, a list for source points (called source-list) was maintained to keep the initial seed point, as well as the vessel bifurcations that were detected during the vessel tracking procedure. In addition, a second list of crossings (called crossing-list) was designed to keep the possible crossings of two vessels. *Then*, a sampling disk with about half the size of the vessel diameter was used in the PTO to automatically track the artery segments (or branches) one at a time, beginning from the seed point and stopping at the vessel termination. During the tracking, the proposed SPD functioned simultaneously to report the local vessel pattern as bifurcation, crossing, segment, or termination. All detected vessel bifurcations were stored one at a time at the top of the source-list and were later used as subsequent starting points for tracking their respective artery segments (or branches). *Finally*, the vessel PTO and the SPD stop tracking and identifying vessels once all vessels have been tracked and registered.

### Tracking criterion

The tracking mechanism was designed based on the criterion of *continuity properties of luminance*, *position*, and *diameter *of the general coronary artery in the angiographic images. The appropriate incorporation of these properties into the PTO enables successful management of the cases of vessel gap (caused by nonuniformity of the contrast medium or inappropriate thresholding in the preprocessing step) and arterial lesion (such as stenosis or aneurysm).

### Description of PTO

Our PTO performs automatically along the coarse vessel path extracted from the vessel feature map. It begins or stops tracking an artery segment (or branch), depending on the structure pattern detected at current artery element *e_t_*. The tracking problem at simple artery positions, with no bifurcations, crossings, or terminations, is to determine the next artery element *e*_*t*+1 _in the vessel path. Each artery element *e_t _*has four associated attributes, namely position **x***_t_*, vessel feature $\overset{\wedge }{Z}({\text{x}}_{t})$, direction vector *ϕ_t _*= **v**_2_(**x***_t_*), and vessel diameter *d_t_*. These attributes guide the vessel tracking algorithm. For efficiency, a sampling disk centered at the current artery element *e_t _*was used to search for a set of candidate elements $\left\{({\text{x}}_{t+1}^{i},\overset{\wedge }{Z}({\text{x}}_{t+1}^{i}),\phi_{t+1}^{i},d_{t+1}^{i})|i\in [1,...,N_{s}]\right\}$ from the overlapping region between the sampling disk and the binarized vessel feature map. From artery element *t *to *t*+1, the probabilistic tracking can be formulated by a maximum posterior probability (MAP), expressed as

(4) $${\overset{\wedge }{\text{x}}}_{t+1}=\arg\underset{i\in [1,...,N_{t}]}{\max}P({\text{x}}_{t+1}^{i}|{\text{x}}_{t})$$

**Figure 3:**
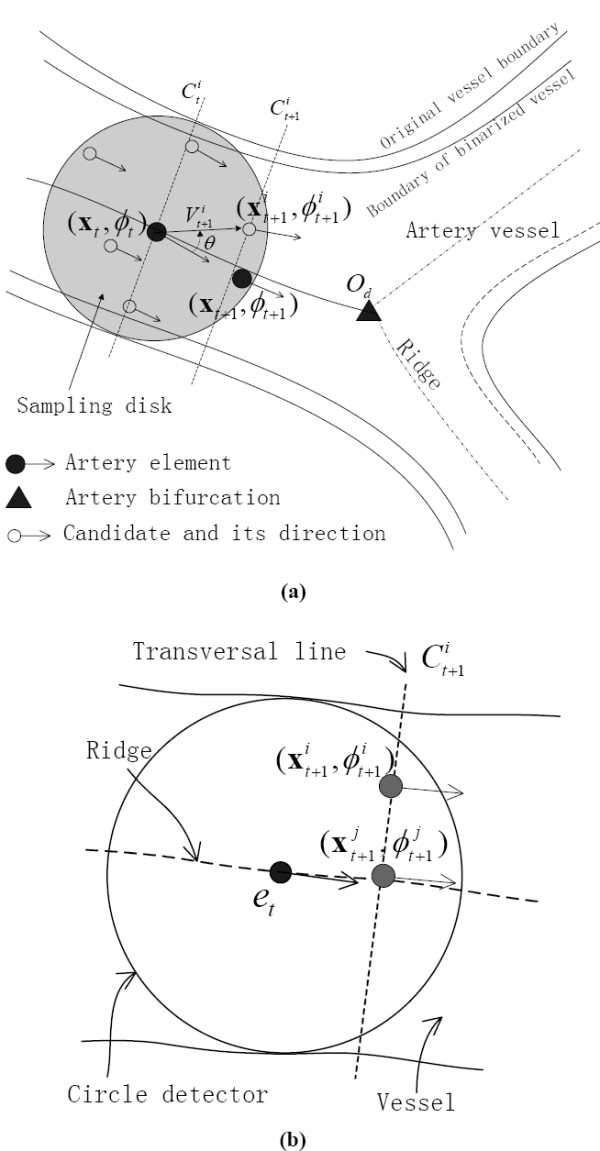
**Show the probabilistic tracking and diameter measurement along vessel path**. (a) is the description of the probabilistic tracking. (b) depicts the estimation of vessel diameter using the vessel feature profile of transversal line $C_{t+1}^{i}$.

The sequent modeling was based on the aforementioned continuity properties of the artery vessel. During tracking vessel length [Figure 3(a)], three local terms were used to constrain the candidates based on vessel feature continuity, longitudinal direction continuity, and diameter continuity. The first term, $\overset{\wedge }{Z}({\text{x}}_{t+1}^{i})$, was acquired from the vessel feature map at candidate position ${\text{x}}_{t+1}^{i}$. The second term to some extent, kept the vessel tracking from being interrupted by noise, pseudo contours, and crossing, although more assurances should come from the structure pattern detection result, as described in aforementioned sections. Given vector $V_{t+1}^{i}$ pointing from current position **x***_t _*to candidate point ${\text{x}}_{t+1}^{i}$, a deflection of vessel direction $\theta_{t+1}^{i}=∠V_{t+1}^{i}-∠\phi_{t}$ [Figure 3(a)] yields a means of the longitudinal continuity. In the case of vessel crossing or paralleling with each other (especially for thin vessels), the third term of diameter continuity enables the tracking of the current vessel segment/branch to proceed without being affected by neighboring vessels. Given $d_{t+1}^{i}$ and *d_t _*as the vessel diameters, respectively at candidate point ${\text{x}}_{t+1}^{i}$ and current artery element **x***_t_*, increment $\nabla d_{t+1}^{i}=|d_{t+1}^{i}-d_{t}|$ yields the measure of diameter continuity. Altogether, the estimation of the optimal candidate gives emphasis to the continuity properties, which is also the basic idea for probabilistic tracking. In the following paragraph, we provide further discussion to mathematically model.

First, the relationship between the sampling instance and the posterior probability can be described as

(5) $$\left(\overset{\wedge }{Z}({\text{x}}_{t+1}^{i});\;\theta_{t+1}^{i}\text{; }\nabla d_{t+1}^{i}\;\right)\;|\;{\text{x}}_{t}\to P({\text{x}}_{t+1}^{i}|{\text{x}}_{t})$$

All feature measures in the left of the abovementioned expression are normalized before participation in the estimation of probability. In particular, maximal vessel feature ${\overset{\wedge }{Z}}_{\max}$ and maximal diameter *d_max _*are determined from all *N_s _*candidate points in the sampling disk, and are used to normalize the respective measures. Note that in practice, estimated vessel direction ∠*ϕ_t _*can be reversed; thus, it should be considered in the normalization of $\theta_{t+1}^{i}$. Given normalized measures $Q_{Z}({\text{x}}_{t+1}^{i})=|1-\overset{\wedge }{Z}({\text{x}}_{t+1}^{i})/{\overset{\wedge }{Z}}_{\max}|$, $Q_{\theta }(v_{t+1}^{i})=\frac{1}{\pi }\min\{|\arg\theta_{t+1}^{i}-\pi |,|\arg\theta_{t+1}^{i}|\}$, and $Q_{d}({\text{x}}_{t+1}^{i})=\nabla d_{t+1}^{i}/d_{\max}$, their probabilities can be respectively written as:

(6.1) $$q_{Z}({\text{x}}_{t+1}^{i})=\frac{1}{\sqrt{2\pi }\sigma_{Z,t}}\cdot \exp\left(\frac{-Q_{Z}^{2}({\text{x}}_{t+1}^{i})}{2\sigma_{Z,t}^{2}}\right)$$

(6.2) $$q_{\theta }({\text{x}}_{t+1}^{i})=\frac{1}{\sqrt{2\pi }\sigma_{\theta ,t}}\cdot \exp\left(\frac{-Q_{\theta }^{2}({\text{x}}_{t+1}^{i})}{2\sigma_{\theta ,t}^{2}}\right)$$

(6.3) $$q_{d}({\text{x}}_{t+1}^{i})=\frac{1}{\sqrt{2\pi }\sigma_{d,t}}\cdot \exp\left(\frac{-Q_{d}^{2}({\text{x}}_{t+1}^{i})}{2\sigma_{d,t}^{2}}\right)$$

where σ*_Z,t_*, σ*_θ,t_*, and σ*_d,t _*are the standard deviations, which can be estimated by calculating the variances of the corresponding measures in all *N_s _*candidates. For example, σ*_Z,t _*is estimated as

(7) $$\sigma_{Z,t}=\sqrt{\frac{1}{N_{s}}\sum_{i=1}^{N_{S}}{\left[Q_{Z}({\text{x}}_{t+1}^{i})-{\bar{Q}}_{Z}\right]}^{2}}$$

where ${\bar{Q}}_{Z}$ is the average measure obtained by ${\bar{Q}}_{Z}=1/N_{s}\sum_{i=1}^{N_{s}}Q_{Z}({\text{x}}_{t+1}^{i})$. By integrating all distribution functions in Eq. (6), the optimal estimation of artery element *e*_*t*+1 _can be obtained by

(8) $$e_{t+1}={\overset{\wedge }{\text{x}}}_{t+1}=\arg\underset{\begin{array}{c} {\text{x}}_{t+1}^{i},\\ i=1,...,N_{s} \end{array}}{\max}(q_{z}({\text{x}}_{t+1}^{i})\cdot q_{\theta }({\text{x}}_{t+1}^{i})\cdot q_{d}({\text{x}}_{t+1}^{i}))$$

PTO uses adaptive parameters adjusted according to Eq. (7) based on all candidates in the sampling disk. Thus, it can successfully estimate the artery elements along the vessel path even in cases of gaps and arterial lesions. The gap is generally regarded as the region of a longitudinal narrow crack with a width less than the vessel radius, whereas the arterial lesion presents as a short or long component of the longitudinal region with large variations in vessel diameter and, at times, weak luminance. During the vessel tracking, the candidates running into the gaps can be crossed out by the continuity terms, thus enabling PTO to continue working. On the other hand, in case candidates run into the regions of the artery lesion, the PTO still carries on, even with large variations in vessel diameter.

### Artery diameter estimation

Considering that the blood vessels have measurable abnormalities in diameter, intensity, and tortuosity, the artery diameter of candidate ${\text{x}}_{t+1}^{i}$ should be estimated adaptively by analyzing its 1-D vessel feature profile orthogonal to the vessel direction. All candidates sitting along the sample profile have the same vessel diameter estimated (as described next). For example, in Figure 3(b), another candidate ${\text{x}}_{t+1}^{j}$ sitting at the peak of the profile has the same vessel diameter as ${\text{x}}_{t+1}^{i}$. The profile can be represented by transversal line $C_{t+1}^{i}$ with a single pixel width, as shown in Figure 3(b). Candidates ${\text{x}}_{t+1}^{i}$ and ${\text{x}}_{t+1}^{j}$ have the same profile, and thus the same diameter.

The feature values in the particular profile can be obtained from vessel feature map $\overset{\wedge }{Z}$. The vessel diameter on this profile can be measured through the convolution of the normalized second derivative of the 1-D Gaussian function with the profile. Standard deviation σ in the 1-D Gaussian function should be adjusted during the vessel diameter estimation. Standard deviation $\overset{\wedge }{\sigma }$ that results in the largest response among all convolution results with the use of different standard deviations is finally selected as the estimated diameter for all candidates sitting on the same profile. Using this method, we can estimate vessel diameter $d_{t+1}^{i}$ for candidate ${\text{x}}_{t+1}^{i}$.

This vessel diameter estimation process proceeds automatically without user interaction. The relevant diameter estimation results are demonstrated in the experimental section.

### Inferring structure pattern of artery vessel

When the PTO works on current artery element **x***_t_*, the SPD should also work in parallel to report the structure pattern to guide the PTO. Our SPD performs in the following manner. First, a circular pattern detector is centered on current artery element **x***_t_*. It then acquires the circular cross-section profile from vessel feature map $\overset{\wedge }{Z}$. Subsequently, it captures local vessel measures, including the number of peaks in profile (M_*t*_), the azimuth vector directing from the detector center to every peak (*p_m_*), and the Gabor response on each peak (*γ_o.m_*). Finally, it returns the structure pattern inferred by a fuzzy inferring operator using the fuzzy membership degrees of multi-feature fuzzy subsets.

### Detecting the peaks of the vessel-feature profile

The pattern detector samples of vessel features $\overset{\wedge }{Z}$ along the circular cross-section profile are centered at current artery element **x***_t _*with radius *r_t _*= *α*·*d_t_*/2. Generally, the range of parameter *α *in *α *∈ [1,1.5] is preferred for better measurement because in this case, the radius of circular pattern detector is a little bit larger than half of vessel diameter *d_t _*of current artery element **x***_t_*; thus, the structure around **x***_t _*can be adaptively and completely captured. The resulting vessel feature profile is called circular template *T*_(**x**,*r,t*)_, which consists of a fixed number (e.g., 150) of vessel features sampled over the circumference of a circle (**x**_*t*_, *r*_*t*_). The same number of vessel features $\overset{\wedge }{Z}$ is sampled to make the SPD adaptive to different sizes of vessel diameters. Therefore, even for small vessels, the same number of points is also sampled. Because vessel feature map $\overset{\wedge }{Z}$ is defined in discrete domain, the bilinear interpolation is used for sampling features from $\overset{\wedge }{Z}$ when generating circular template *T*_(**x**,*r,t*)_.

**Figure 4:**
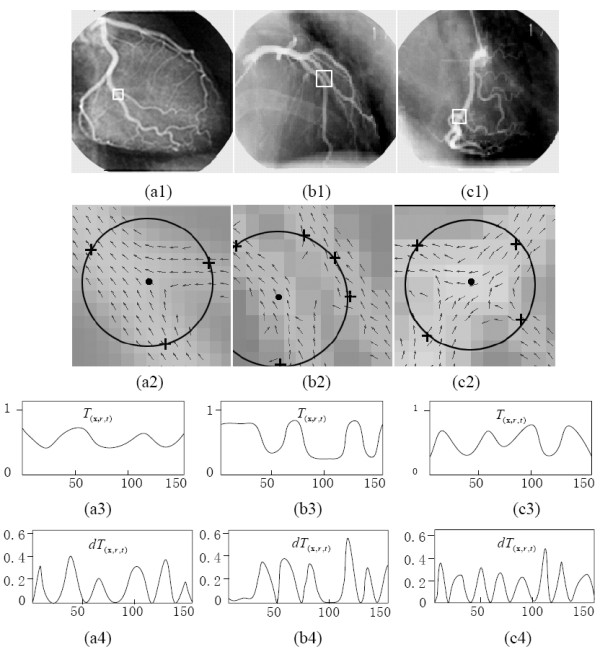
**Using the circular pattern detector to characterize the vessel structures, in the cases with bifurcation, paralleling, and crossing patterns**. (a1)-(c1) are original images and the respective regions of interest. (a2)-(c2) are the corresponding direction maps overlaping with circular pattern detectors. (a3)-(c3) are the corresponding vessel-feature profiles on the circular template *T*_(**x**,*r,t*)_. (a4)-(c4) are the corresponding magnitude profiles of the first-order derivative *dT*_(**x***,r,t*)_.

The sampling process is demonstrated in Figure 4, in which the vessel feature profiles are sampled by the circular templates (*T*_(**x**,*r,t*)_) in 4(a2)-4(c2). The typical profiles are shown in 4(a3) - 4(c3). To detect the peaks in each profile, first-order derivative *dT*_(**x**,*r,t*) _of *T*_(**x**,*r,t*) _is computed through the circular convolution of *T*_(**x**,*r,t*) _with the first-order derivative of the 1-D Gaussian filter. Figures 4(a4) - 4(c4) shows the magnitude profile of the resulting first-order derivative |*dT*_(**x**,*r,t*)_|. The local minima of the derivative magnitude correspond to the maxima or minima of the vessel feature profile along *T*_(**x**,*r,t*)_. The peaks of the profile corresponding to maxima can be detected according to the derivative signs. For example, a peak of the profile corresponds to the sign transition of the first-order derivative from positive to negative. In this manner, we can detect all peaks along with their locations [e.g., z{*p_m _*= (*x_m_*, *y_m_*), *m *= 1,...,*M_t_*}], where *M_t _*is the total number of peaks.

The resulting peaks provide the SPD vessel with several important properties, such as their positions, vessel features, vessel directions, as well as their relationships with the center of pattern detector (**x***_t_*). To better infer the pattern of the vessel structure, more relevant metric features, such as the Gabor features described in the next section, should be extracted.

### The Gabor responses at locations of the peaks

Considering that the peaks of the circular profile provide useful information for structure identification, we first discuss how to infer the case in which two points (apart less than a few pixels) are located at the same vessel axis line. This case may also be called "co-vessel." To detect whether peak point *p_m _*= (*x_m_*, *y_m_*) and detector center (*O_d_*) are located in the same segment of the vessel, the Gabor filter operator [14,15] was used for local description. As a function of orientation, frequency, and scale, the operator can characterize the dominant attributes at peak point *p_m _*= (*x_m_*, *y_m_*). Note that this responds strongly to the isolated vessel strip, but exhibits a weaker or no response to the noise.

**Figure 5:**
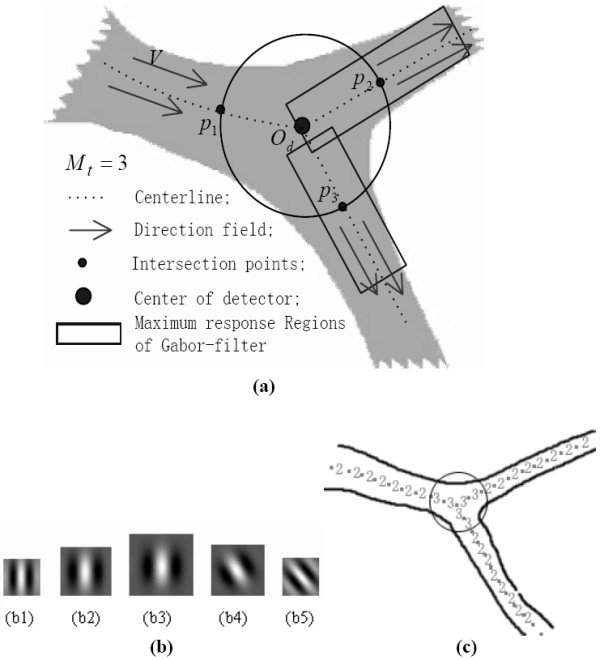
**The feature integration, Gabor masks, and structure detection near artery bifurcation**. (a) illustrates circular pattern detector and feature integration. (b) is an examples of five Gabor masks, and their parameters, where (b1) *T*_1 _= 5, *θ*_1 _= *π*/2, *f*_1 _= 0.2; (b2) *T*_2 _= 9, *θ*_2 _= *π*/2, *f*_2 _= 0.111; (b3) *T*_3 _= 13, *θ*_3 _= *π*/2, *f*_3 _= 0.077; (b4) *T*_4 _= 9, *θ*_4 _= 3*π*/4, *f*_4 _= 0.111; (b5) *T*_5 _= 5, *θ*_5 _= 3*π*/4, *f*_5 _= 0.2. (c) illustrates the problem of the uncertainty of the node positions.

Given the relationship between the Gabor filter scale and the frequency with 2σ = 2*πf *= *T*, several masks by the Gabor operator can be defined by the real component of the Gabor function with a size of *T *× *T*. The masks of different scales are shown in Figure 5(b). Thus, to characterize local attributes, such as direction and "co-vessel" at a peak point, we merely need to compute the responses of several Gabor masks, and retain only the related parameters corresponding to the maximum response. Although none of these parameters are invariant to common image transformation, such as scaling and rotation, only one set of parameters corresponds to maximum response *g*_max _at the considered position. Given vector *V_o,m _*directing from the detector center to the peak position (*x_m_*, *y_m_*), the following response measure *γ_o,m _*represents the degree with which peak *p_m _*= (*x_m_*, *y_m_*) belongs to the same vessel axis line of the center point of the pattern detector

(9) $$\gamma_{o,m}=\frac{1}{g_{\max}}\arg{\text{ max}}_{\sigma }|G_{∠V_{o,m},\sigma /\pi ,\sigma }({\text{x}}_{m},y_{m})|$$

Where 0 <*γ_o.m _*< 1. With an image sized 512 × 512, the size of the mask (σ) is equal to the vessel diameter, which ranges from 3 to 14 pixels. Therefore, the resulting measure *γ_o,m _*reflects the relationship between peak *p_m _*and the detector center. For example, the resulting measure *γ_o,m _*at peak *p_m _*is lower if mask orientation *V_o,m _*does not accord with the local vessel direction, that is, there is no "co-vessel" existing between the peak point and the detector center.

### The multi-feature based fuzzy inferring function (FIF)

Four types of patterns can be inferred, namely, vessel termination (*i *= 1), artery segment (*i *= 2), bifurcation (*i *= 3), and crossing point (*i *= 4), where variable *i *denotes the index for the ordinal structure pattern of the artery vessel. With effects from noise and pseudo edge, the number of peaks in each of these four types of patterns might be higher than the actual one. For example, by comparing the circular cross-section profile of different cases (Figure 4), we can observe that more than two peaks might occur in the profile of the artery segment, and more than three or four peaks might occur in the profile of the bifurcations or crossings, respectively. Without loss of generality, the circular pattern detector detects the number of peaks from the ordinal structure pattern according to *M_t _*≥ 1, *M_t _*≥ 2, *M_t _*≥ 3 and *M_t _*≥ 4 [Figure 5(a)]. Thus, the number *M_t _*can be regarded as a pseudo pattern of the artery structure. On the other hand, each peak *p_m _*has two metric features important to pattern identification: vessel feature $\overset{\wedge }{Z}(p_{m})$ at peak *p_m_*, and response measure *γ_o,m _*of Gabor mask function $G_{∠V_{o,m},\sigma /\pi ,\sigma }(x_{m},y_{m})$. The relationship between the pattern detector and the metric features for a typical artery bifurcation is shown in Figure 5(a).

In the following section, the fuzzy subsets are defined for all metric features, as well as for number of peaks *M_t_*. They will then be integrated for pattern inferring of point **x***_t_*.

***Definition *1: **In the region $\Omega =\{p_{m},{\overset{\wedge }{Z}}_{m},\gamma_{o,m},M_{t}|1\leq m\leq M_{t};{\overset{\wedge }{Z}}_{m},\gamma_{o,m}\in [0,1]\}$, given that fuzzy subsets $F_{m,\overset{\wedge }{Z}}$ and *F_m,γ _*correspond to the metric features of peak *p_m_*, and fuzzy subset $F_{M_{t},{\text{x}}_{t}}^{i}$ corresponds to the *i*-th pseudo pattern at current position **x***_t_*, the membership degrees of these fuzzy subsets can be defined by the standard Gaussian function as

(40) $$\begin{array}{l} \mu_{m,\overset{\wedge }{Z}}=\exp\left(\frac{{\left({\overset{\wedge }{Z}}_{\max}-{\overset{\wedge }{Z}}_{m}\right)}^{2}}{-2\sigma_{Z}^{2}}\right);\mu_{m,\gamma }=\exp\left(\frac{{\left(\gamma_{\max}-\gamma_{o,m}\right)}^{2}}{-2\sigma_{\gamma }^{2}}\right);\\ \;\mu_{M_{t},{\text{x}}_{t}}^{i}=\{\begin{array}{ll} \exp\left(\frac{{\left(M_{t}-i\right)}^{2}}{-2\sigma_{M_{t}}^{2}}\right) & M_{t}\geq i\\ 0 & \text{else} \end{array} \end{array}$$

where. ${\overset{\wedge }{Z}}_{\max}=\max_{m}{\overset{\wedge }{Z}}_{m}$., *γ*_max _= max_*m *_*γ*_*o,m*_, and *m *= 1,...,*M_t_*. The above expressions, $\mu_{m,\overset{\wedge }{Z}}$ and *μ_m,γ_*, represent the respective degrees of membership of two metric features at current position **x***_t_*, which are used to ensure that each peak *p_m _*is located at the center of the vessel. Meanwhile, $\mu_{M_{t},{\text{x}}_{t}}^{i}$ provides the membership degree of the pseudo pattern based on the number of peaks. According to the abovementioned membership degrees, the structure patterns of current position **x***_t _*can be inferred by the following definition.

***Definition *2: **In the same region Ω, given *J_i_*(**x***_t_*) the fuzzy subset of the structure pattern belonging to the *i*-th pattern at current position **x***_t_*, the relationship between fuzzy set *J_i_*(**x***_t_*) and the fuzzy sets of the metric features is given by

(11) $$J_{i}({\text{x}}_{t})=\{\overset{M_{t}}{\underset{m=1}{\text{I}}}F_{m,\overset{\wedge }{Z}}\}\text{I}\{\overset{M_{t}}{\underset{m=1}{\text{I}}}F_{m,\gamma }\}\text{I}\{F_{M_{t},{\text{x}}_{t}}^{i}\}$$

The membership degree of *J_i_*(**x***_t_*) is given by:

(12) $$\mu_{J}^{i}({\text{x}}_{t})=(\overset{M_{t}}{\underset{m=1}{\wedge }}\mu_{m,\overset{\wedge }{Z}})\wedge (\overset{M_{t}}{\underset{m=1}{\wedge }}\mu_{m,\gamma })\wedge \mu_{M_{t},{\text{x}}_{t}}^{i}$$

where it is shown that the direct inferring of the local vessel structure can be hardly implemented using $\mu_{J}^{i}({\text{x}}_{t})>T_{\alpha }$ with threshold *T_α_*. Therefore, the optimal pattern inference should be based on the principle of maximum membership degree and can be expressed as

(13) $$\mu_{J}^{i0}({\text{x}}_{t})=\overset{4}{\underset{i=1}{\vee }}[\mu_{J}^{i}({\text{x}}_{t})]$$

which infers the artery structure at current position **x***_t _*belonging to the *i*_0_-th pattern.

When the detector center is near the vessel node (e.g., bifurcation or crossing), the resulting node position can be ambiguous; for example, a few of the redundant positions may possibly be the node positions because of the similarity of their metric features to those of the actual node. To solve this problem, a second local maximum operation is performed to obtain a preferable node position. Given *n *redundant positions near the bifurcation, with the membership degrees $\left\{\mu_{J}^{i_{3}}({\text{x}}_{t}^{1}),...,\text{ and }\mu_{J}^{i_{3}}({\text{x}}_{t}^{n})\right\}$, the preferable one should be located at

(14) $$\overset{\wedge }{\text{x}}=\arg\underset{{\text{x}}_{t}^{k},k=1,...,n}{\max}(\mu_{J}^{i_{3}}({\text{x}}_{t}^{k}))$$

In Figure 5(c), the numbers 2 and 3 represent the two structure patterns determined in the corresponding locations by the proposed pattern detector. Pattern detection result is used to guide the vessel tracking process.

### Manipulation of vessel tracking process and organiztion of tracking results

Appropriate termination conditions for vessel tracking are important to achieving better tracking performance. Our vessel tracking algorithm stops tracking the current artery segment or branch once one of the following conditions is satisfied: (1) a new vessel bifurcation is detected, which should be dealt with appropriately; (2) vessel feature $\overset{\wedge }{Z}({\text{x}}_{t+1})$ at position **x**_*t*+1 _along the vessel path is below a certain threshold, such as when a value is slightly higher than the background (this threshold is set as 0.05 in this paper); (3) the current path overlaps with a vessel path previously detected, except when a crossing point on the vessels has been detected and recorded in advance; (4) the source-list is empty and no new node is found; and (5) the new point is outside the image field. In all cases except for vessel crossing, the last valid vessel point is marked as a termination point, and the algorithm starts tracking the other vessel with the seed point placed at the top of the source-list.

The artery segment is a vessel length with two end-points at two nodes, or at an origin point and a node, whereas the artery branch is a distal vessel with a distal point. Either the artery segment or branch is composed of artery elements. Considering these artery elements arranged at intervals (or with "look-ahead distance") of less than the radius of the sampling disk, polylines are used to connect the artery elements by linear interpolation. The generated polylines are treated as an approximation of vessel centerlines, and have high probability inside the binarized vessel structures.

## Results

The proposed algorithm was tested on both simulated images and real image sequences with stenosis vessel and aneurysm. To obtain images with acceptable quality, the cranial and caudal angles, as well as the X-ray dose, were adjusted so that the X-rays travel a moderate distance. Our vessel tracking algorithm was implemented in MATLAB 7.0 on a Pentium IV PC (with CPU 2.8 G and 512 M memory).

Our algorithm was first tested on various simulated artery vessel images to adjust its parameters. Actual images were used to evaluate the actual performance of the algorithm by comparing the algorithm-segmented structure and vessel length of the entire artery network with those produced by expert cardiologists. The tracking algorithm has several distinguishing characteristics: (a) it is robust to the starting position detected; (b) it can detect and identify a larger area of the artery tree without any manual intervention; and (c) it can accurately distinguish the narrows resulting from the gaps in the vessel feature map or those caused by real vessel stenosis.

In our algorithm, total computational time *T *consisting of two major components *T*_1_, *T*_2_, and one minor component *T*_3, _were spent on preprocessing (36.7 s), tracking (15.7 s), and connecting artery elements (1.4 s), respectively.

General effects of vessel segmentation are listed in the first column of Table 1, where the results of right coronary artery (RCA) and the left coronary artery (LCA) in the real angiographic images are compared separately. In Table 1, *L*_∑ _and $\bar{L}$ denote the length of algorithm-segmented vessels (in pixels) and the total vessel length delineated by expert cardiologists, respectively. The percentage of the detected artery segments (DAS %) and the extracted vessel length (EVL %), as well as the identification ratio (IR %) of the arterial structure was computed to evaluate the performance of our algorithm. In particular, the IR % is defined as the percentage of the correct parts of vessel length in the entire length of algorithm-segmented vessels. As openly reported in literature, the DAS % produced by Haris et al. [16] reached 90%, while the EVL % produced by Schrijver et al. [4] was less than 78%. On average, our algorithm can reach 94.7% for DAS % and 88.1% for EVL %.

**Table 1:** Statistical results on artery tracking and structure identification in real angiographic images.

Images, Num.	EVL(%)	IR(%)	$\bar{L}$	*L*_∑_	Average identification rate of bifurcation $\left({\bar{N}}^{b}/{\bar{W}}^{b},i=3\right)$						Average identification rates of different patterns (%)			
					*b *= 1	b = 2	b = 3	b = 4	b = 5	Total	*i *= 1	*i *= 2	*i *= 3	*i *= 4
SAT, 1	99.8	99.6	950	948	1/1	2/2	2/2	3/4	0/0	8/9	100	100	88.9	75

LCA, 128	87.2	98.3	1868	1629	1.00/1.00	2.00/2.00	7.11/7.22	5.13/6.12	0.32/1.34	15.56/17.68	98.9	98.7	88.0	72.8
RCA, 32	91.7	99.1	947	868	1.00/1.00	3.10/3.15	2.65/2.70	0.30/0.33	0.01/0.02	7.05/7.20	98.3	99.5	97.9	77.5
Mean	88.1	98.5	1677	1477	1.00/1.00 (100%)	2.22/2.23 (99.6%)	6.22/6.32 (98.4%)	4.16/4.96 (83.9%)	0.26/1.08 (24.1%)	13.86/15.58 (89.0%)	98.8	98.9	90.0	73.7

Note: *L*_∑ _and $\bar{L}$ denote the length of algorithm-segmented vessels and the total vessel length, respectively. *N^b^*/*W^b ^*denotes the rate of bifurcation identification., where *N^b ^*denotes the number of correctly detected bifurcations for the *b*-th level vessel branch, while *W^b ^*denotes the total number of the detected bifurcations.

Other detailed experiments and performances are introduced in the next section.

### Arterial vessel simulation and algorithm evaluation

**Figure 6:**
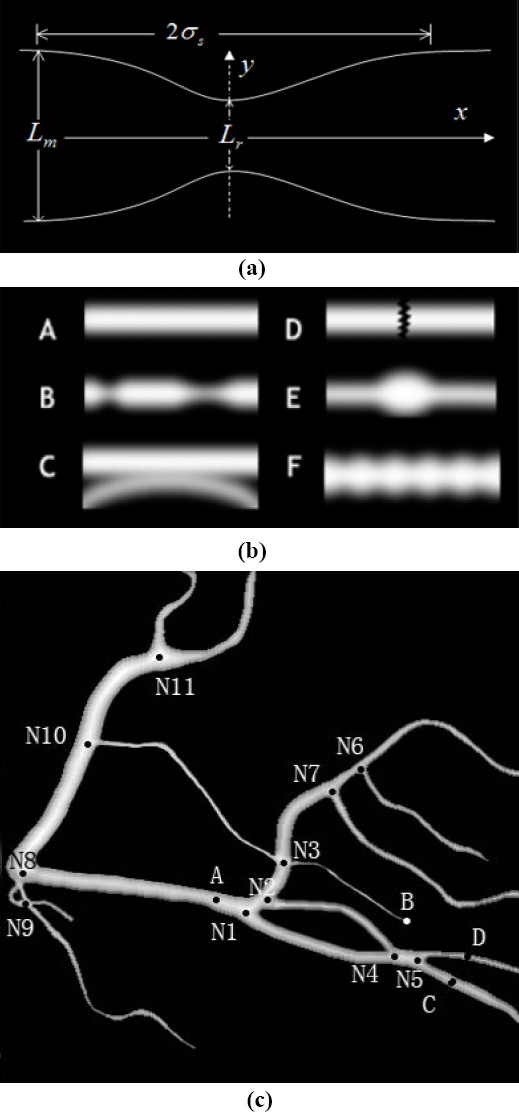
**The simulations of arterial vessels: (a) the local coordinate system of vessel simulation; (b) the simulated arterial segments; (c) the SAT**.

Simulated data were created for the arterial segment [Figure 6(b)] and the simulated arterial tree (SAT) [Figure 6(c)]. In particular, various levels of Gaussian smoothing were applied to simulate the spatial blurring in an X-ray projection. The SAT in Figure 6(c) was generated to simulate artery characteristics, such as curving, overlapping, and bifurcating. For these simulated images, all information related to vessel segments, centerline, and local structure are known beforehand. We can analyze the algorithm characteristics given that various levels of white Gaussian are added to these images.

The performance of our algorithm can first be evaluated using the simulated images, such as vessel segments A to F. Given an initial seed point at the left end of the vessel segment in A, B, E, and F of Figure 6(b), the PTO can automatically track vessels and measure their diameters. The measured diameters along the vessel are consistent with varying morphology, such as stenoses and aneurysms. The resulting mean errors are listed in Table 2, where *L_x _*denotes the length of the simulated arterial segment, *e*_0 _and *e_d _*denote the mean error of the resulting vessel centerlines and diameters, respectively. Under zero-level Gaussian noise, the mean errors of estimated centerlines and diameters of stenosis or aneurysm vessels are no more than 0.02 and 0.04, respectively. In case of close proximity (such as C) and local gap (such as D), the tracking results depend on distance *d_P _*between two neighboring vessels and gap-width *w_G_*, respectively. Suppose that *r*_1 _and *r*_2_, and *r*_D _denote the radii of two neighboring vessels and the radius of the detector, respectively. To enable the algorithm to operate through the vessel despite influence from the neighboring vessel and the gaps, the limiting distance and gap-width would be *d_P _*≥ (*r*_1 _+ *r*_2_)/2 and *w_G _*<*r_D_*, respectively. The entire length of the skeleton lines of the SAT is 948 pixels, and is entirely involved in the testing.

**Table 2:** Parameters and mean errors in testing of the phantoms of arterial segments.

	A	B	C	D	E	F
*S_V_*	1	1	1	1	1	1
*S_B_*	0	0	0	0	0	0
σ*_B_*	3.2	3.2	3.2	3.2	4.0	3.2
*L_m_*	13	13	13, 6	13	9	15
*L_r_*	13	5	13, 6	13	18	10
*L_x_*	120	120	120, 125	120	120	120
σ*_w_*	0	0	0	0	0	0
*e*_0_	0.01	0.01	0.01,0.02	0.01	0.02	0.02
*e_d_*	0.02	0.04	0.05,0.05	0.02	0.04	0.03

The results of structure identification on the SAT and real angiograms are provided in the following sections.

### Experiments on structure pattern inferring

Two experiments were performed on simulated and real images to test the capability of our algorithm to infer the structure patterns in the artery tree. The performances on identification of all four different patterns, as well as separate performances on the detection of bifurcation are reported.

**Figure 7:**
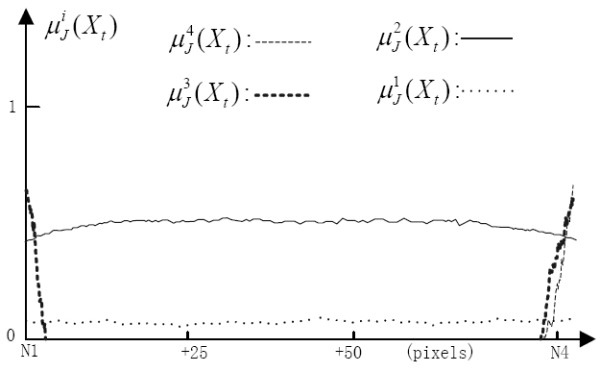
**The fuzzy memberships measured along the vessel axis, starting from point N1 to N4 in the SAT**.

The first experiment was conducted on the SAT image. Based on results of estimated membership degrees $\mu_{J}^{i}(X_{t})$ in Eqs. (13) and (14), the attributes of all interesting points in Figure 6(c) can be well characterized by our algorithm. Overall, the captured membership degrees corresponded well to four different structure patterns, such as the membership degree distribution between N1-**N4 **in Figure 7. For vessel overlapping cases, such as local superposition between the two vessel segments N2-**N4***-***N5**-D and N1-**N4**-**N5**-C, the structure ambiguity (between **N4 **and **N5**) might be difficult to resolve using only a single-view projection, although it might be resolved by observing vessel motion or radius information from sequence images as done by human observers while reading cine-angiograms. In short, our multi-feature based fuzzy inferring method is capable of accurately estimating the structure patterns from 2D images.

The second experiment was performed on actual angiogram images selected from the angiogram sequences. The related results are provided in the second and third columns of Table 1. The lower level of the arterial structure can be inferred with higher IR %, and the recognizable vessels can reach up to the fifth level at best. In practice, the cardiovascular surgeon is often interested in the vessels below the fourth level; thus, the performance of our algorithm is promising. The second column of Table 1 also displays the rate of bifurcation identification (*N^b^*/*W^b^*). Here, *N^b ^*denotes the number of correctly detected bifurcations for the *b*-th level vessel branch, while *W^b ^*(≥*N^b^*) denotes the total number of the detected bifurcations, including the incorrectly detected ones.

### Experiments on vessel tracking and measurement

**Figure 8:**
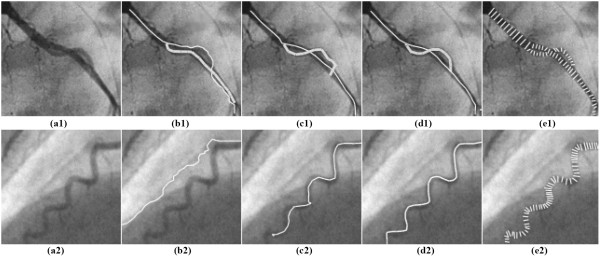
**Comparison of the results of vessel-tracking using the PTO based on different continuity terms**. (a1) & (a2) two original images. (b1) & (b2) using the PTO simply based on *q_θ_*. (c1) & (c2) using the PTO based on *q_θ _*and *q_z_*. (d1) & (d2), using the PTO based on complete terms. (e1) & (e2), measuring the vessel diameters, respectively along the vessel paths in (d1) and (d2).

We also evaluated the respective contributions of the probability terms of the continuity properties given in Eq. (6) to the vessel tracking. By rewriting Eq. (8) as ${\overset{\wedge }{\text{x}}}_{t+1}=f(q_{\theta },q_{Z},q_{d})$, three different types of PTO, namely, ${\overset{\wedge }{\text{x}}}_{t+1}=f(q_{\theta })$, ${\overset{\wedge }{\text{x}}}_{t+1}=f(q_{\theta },q_{Z})$, and ${\overset{\wedge }{\text{x}}}_{t+1}=f(q_{\theta },q_{Z},q_{d})$, can be constructed. Their respective results are compared in Figure 8, where the paths traced in the same sub-image were obtained by the same PTO. Figures 8(b1) and 8(b2) show that the direction constraint term in Eq. (6.2) can effectively compel the sampling disk and the pattern detector to move ahead, but it can often be lost in edges, pseudo contours, neighboring vessels, or even noise. By incorporating the vessel feature term, that is, using ${\overset{\wedge }{\text{x}}}_{t+1}=f(q_{\theta },q_{Z})$, the vessel tracking result can be improved, as shown in Figures 8(c1) and 8(c2). Using all terms together, that is, using ${\overset{\wedge }{\text{x}}}_{t+1}=f(q_{\theta },q_{Z},q_{d})$, the vessel tracking result can be further improved; this is reflected as the smooth and accurate tracking paths detected in Figures 8(d1) and 8(d2). In addition, if only the vessel feature or diameter term is used, the tracking model almost stops near the initial seed position. These visual results are not shown here.

Figures 8(c1) and 8(c2) also yield a typical case in which the algorithm can successfully manage the close proximity or crossing of vessels, as well as varying vessel curvatures, which validates a number of attractive characteristics of the PTO. For example, the algorithm can run across the local vessel gap, proceed along the current vessel branch in spite of crossing, and successfully manage arterial stenoses and aneurysms.

The *diameter *estimation errors at the artery elements are less than 0.23 pixels on average. The results of the axis line estimation along the artery segments in the SAT image indicate that more errors appear near the nodes, with 1.36 pixels on average. These results show that the axis line estimation error at the nodes is controlled within a limited extent. For actual angiograms, we compared the automatically extracted arterial axis lines with the standard arterial centerlines delineated by an expert cardiologist. We also obtained good results, with a mean error of 0.17 pixels and the maximum error close to half of the respective vessel radius. These errors are produced by noise, as well as large curvatures or abnormal morphologies at the locations of vessel nodes.

#### Vessel stenosis

**Figure 9:**
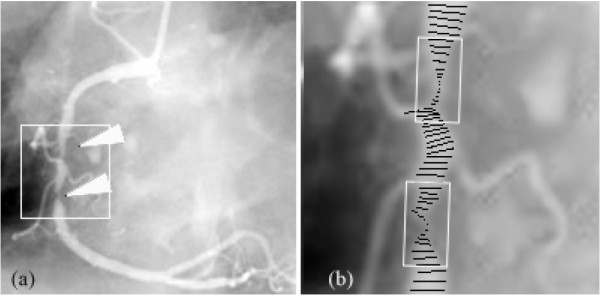
**Tracking and measuring vessel width using the real angiographic images with arterial stenosis**. (a) Original angiogram with arterial stenosises; (b) Location and measurement of the stenosises.

We tested a simple method to determine the position and severity of coronary artery stenosis in the given images, which can be used as an additional evaluation criterion for our tracking algorithm. Our algorithm was able to detect the arterial stenosis. An example in which the diameter of arterial stenosis is tracked and measured is shown in Figure 9. According to stenosis evaluation using the grading scheme of the visual analysis of the American Heart Association [17], %-LENGTH stenosis is estimated as %-LENGTH stenosis = (1-*L_r_*/*L_m_*) × 100%, where *L_r _*and *L_m _*are the estimates of the minimal diameter of the stenotic part of a vessel and the mean diameter of the nonstenotic part, respectively. During tracking and detection, the vessel length of interest begins or terminates when %-LENGTH is 25% higher or lower than the minimum classified value. The vessel length of interest can be described by the rectangular sash in Figure 9.

In the testing, 35 angiograms with 40 stenosis positions were used to evaluate the correctness of stenosis identification, in which all the standard vessel stenoses, as well as their severities were provided beforehand by the expert cardiologist. As a result, 33 stenosis objects are correctly detected and the number of false negatives and false positives are seven and one, respectively. The average correctness rate of stenosis severity identification reaches 68.3%, in comparison with the given stenosis severity levels.

## Discussion

In this paper, we analyzed image data and vessel structures within X-ray projections. A structure distinction exists between the crossing and bifurcation of vessel segments. Two crossing vessel segments have no spatial structure connection, whereas vessel bifurcations (which in general produce three vessel segments) have a structural connection among them. Vessel bifurcation enables low-level vessel segment separation to two high-level ones at the bifurcation point where these vessel segments have different features in their diameters and direction continuity. However, in single-view X-ray projection, two spatial overlapping vessel segments may appear connected to each other at a crossing-point, where the vessel segments maintain their respective features of diameter and direction continuity near the point. The abovementioned vessel continuity of different cases can address, in part, 2D structure ambiguity.

In our algorithm, three parameters can be adjusted to achieve better segmentation results. These include threshold value *r_λ _*in the vessel function Eq. (2), the radius of sampling disk *r_S_*, and the radius of pattern detector *r_D_*. These parameters can be adjusted according to the overall performance integrated from the three percentages, DAS %, EVL %, and IR %. For instance, parameter *r_λ _*may be optimized according to

limrλ→rλ'ΔDAS(rλ)+ΔEVL(rλ)+ΔIR(rλ)Δrλ⋅(DAS(rλ)+EVL(rλ)+IR(rλ))→0

The optimization for parameters *r_S _*and *r_D _*can be conducted similarly. Using the simulated images with different levels of noise, we can determine the optimal values for these three parameters. In the absence of noise, *r_λ _*is computed as $r_{\lambda }=-\sqrt{2\pi \sigma_{f}}$

; parameters *r_S _*and *r_D _*turn out to be equal, that is, *r_S _*= *r_D_*, and are both equal to current diameter *d_t _*of the vessel under tracking.

In the experiments, the time spent in tracking depends on the length of the detected vessel, look-ahead distance *s_t_*, and vessel diameter *d_t_*. Look-ahead distance *s_t _*is determined by the diameter of the sampling disk, which is designed to vary with the diameter of current vessel *d_t_*. The ranges of these parameters are 1 ≤ *s_t _*≤ 7 (pixels) and 3 ≤ *d_t _*≤ 15 (pixels), respectively, which agree with the required relationship 1 ≤ *s_t _*≤ *d_t_*/2. Similarly, more artery elements can result in more computational time.

The rate of bifurcation identification (*N^b^*/*W^b^*) in the second column of Table 1 is related to EVL % and independent of IR % because the IR% is calculated only by the detected vessel points. In addition, the IR % of bifurcation and crossing are relatively lower than that of other structure patterns, which is mostly caused by the structure ambiguity in the 2D image acquisition. According to the testing on SAT, the error ratio is correlated with the effects of image noise and the diameter of the detector.

Based on the results on 40 stenosis objects, the application for the diagnosis of stenosis severity levels do not appear to be very reliable because of the use of single-view projection images. The accurate quantification and analysis of stenotic lesions is actually very complicated and has been elaborately discussed by Sato et al. in [18]. The importance of designing an acquisition system for acquiring "good" images, in which stenotic lesions can be displayed clearly, rather than simply analyzing the given images by common clinic systems, is therefore important.

## Conclusions

We developed a vessel tracking framework for the segmentation and measurement of the arterial tree in angiographic images, and applied it to actual clinical data. Based on the various evaluation results using both simulated and actual data, our algorithm demonstrates a very impressive performance in tracking and measuring artery trees. The particular advantages include efficient handling of vessel nodes, such as bifurcation or crossing; distinguishing between bifurcations and crossings, and automatically locating their positions; and automatic adaptation to varying vessel diameters in the coronary artery. These advantages were made clear with the introduction of our tracking strategies.

Our algorithms are able to locate stenosis or aneurysm positions in a number of given images. Naturally, the actual 3D morphology cannot be simply inferred through the 2D structure patterns. The use of 2D angiograms can pose ambiguity in determining vessel connectivity, particularly in cases with vessel overlapping. This indicates the importance of extending our algorithm to 3D applications to achieve certain diagnostic functions. Moreover, although our fuzzy inferring function is found relatively effective in integrating spatial characteristics of centerline, orientation, diameter, and density along the coronary blood vessels, the performance of structure pattern identification might be further improved, if we can effectively employ advanced learning-based methods, such as support vector machine, to identify the vessel structure patterns. These ideas will be explored further in our future studies.

## Competing interests

The authors declare that they have no competing interests.

## Authors' contributions

ZS carried out the studies on image pre-processing, modeling of vascular structure inferring and tracking, and drafting the manuscript. CW conceived of the study, and participated in its design and coordination, and helped in the drafting of the manuscript. YJ carried out the data acquisition and simulated the coronary artery tree. WY checked the paper writing. All authors read and approved the final manuscript.

## References

[B1] Kawarada O Yokoi Y Morioka N Nakata S Higashiue S Mori T Iwahashi M Hatada A Carotid stenosis and peripheral artery disease in Japanese patients with coronary artery disease undergoing coronary artery bypass grafting Circ J 2003 67 1003 1006 10.1253/circj.67.1003 14639014

[B2] Jian Y Yongtian W Yue L Songyuan T Wufan C Novel Approach for 3-D Reconstruction of Coronary Arteries From Two Uncalibrated Angiographic Images IEEE Trans Image Process 2009 18 7 1563 1572 10.1109/TIP.2009.2017363 19414289

[B3] Kirbas C Quek FKH A review of vessel extraction techniques and algorithms ACM Comput Surv 2004 36 2 81 121 10.1145/1031120.1031121

[B4] Schrijver M Slump CH Automatic segmentation of the coronary artery tree in angiographic projections Proceedings of ProRISC 2002 449 464

[B5] Behrens T Rohr K Stiehl HS Robust segmentation of tubular structures in 3-D medical images by parametric object detection and tracking IEEE Transactions on Systems, Man, and Cybernetics - Part B, Cybernetics 2003 33 4 554 561 10.1109/TSMCB.2003.814305 18238205

[B6] Olabarriaga SD Breeuwer M Niessen WJ Minimum cost path algorithm for coronary artery central axis tracking in CT images In proceedings of MICCAI 2003 2879 687 694 LNCS

[B7] Al-Kofahi KA Can A Lasek S Szarowski DH Dowell-Mesfin N Shain W Turner JN Roysam B Median-based robust algorithms for tracing neurons from noisy confocal microscope images IEEE Trans Inf Technol Biomed 2003 7 4 302 17 10.1109/TITB.2003.816564 15000357

[B8] Wink O Niessen WJ Viergever MA Multiscale vessel tracking IEEE Trans Med Imaging 2004 23 1 130 133 10.1109/TMI.2003.819920 14719694

[B9] Tolias YA Panas SM A fuzzy vessel tracing algorithm for retinal images based on fuzzy clustering IEEE Trans Med Imaging 1998 17 2 263 273 10.1109/42.700738 9688158

[B10] Lorenz C Carlsen I-C Buzug TM Fassnacht C Weese J Multi-scale line segmentation with automatic estimation of width, contrast and tangential direction in 2-D and 3-D medical images CVRMED-MRCAS'97 1997 1205 233 242 full_text

[B11] Sato Y Nakajima S Shiraga N Atsumi H Yoshida S Koller T Gerig G Kikinis R 3D multi-scale line filter for segmentation and visualization of curvilinear structures in medical images Medical Image Analysis 1998 2 2 143 168 10.1016/S1361-8415(98)80009-1 10646760

[B12] Frangi AF Niessen WJ Vincken KL Viergever MA Multiscale vessel enhancement filtering Medical Image Computing and Computer-Assisted Intervention - MICCAI'98 1998 1496 130 137

[B13] Krissian K Malandain G Ayache N Vaillant R Trousset Y Model based detection of tubular structures in 3-D images Computer Vision and Image Understanding archive 2000 80 130 171 10.1006/cviu.2000.0866

[B14] Moreno P Bernardino A Santos-Victor J Model based selection and classification of local features for recognition using Gabor filters Lecture Notes in Computer Science, Image Analysis and Recognition 2006 4142 181 192 full_text

[B15] Moreno P Bernardino A Santos-Victor J Gabor parameter selection for local feature detection Lecture Notes in Computer Science, Pattern Recognition and Image Analysis 2005 3522 11 19

[B16] Haris K Efstratiadis SN Maglaveras N Pappas C Gourassas J Louridas G Model-based morphological segmentation and labeling of coronary angiograms IEEE Trans Med Imaging 1999 18 10 1003 1015 10.1109/42.811312 10628959

[B17] Austen WG Edwards JE Frye RL Gensini GG Gott VL Griffith LS McGoon DC Murphy ML Roe BB A reporting system on patients evaluated for coronary artery disease Circulation 1975 51 4 5 40 1116248 10.1161/01.cir.51.4.5

[B18] Sato Y Araki T Hanayama M Naito H Tamura S A viewpoint determination system for stenosis diagnosis and quantification in coronary angiographic image acquisition IEEE Trans Med Imaging 1998 17 1 121 137 10.1109/42.668703 9617913

